# Fat mass- and obesity-associated gene *Fto* affects the dietary response in mouse white adipose tissue

**DOI:** 10.1038/srep09233

**Published:** 2015-03-18

**Authors:** Justiina Ronkainen, Tuija J. Huusko, Raija Soininen, Eleonora Mondini, Francesca Cinti, Kari A. Mäkelä, Miia Kovalainen, Karl-Heinz Herzig, Marjo-Riitta Järvelin, Sylvain Sebert, Markku J. Savolainen, Tuire Salonurmi

**Affiliations:** 1 Biocenter Oulu, University of Oulu, Oulu, Finland; 2 Institute of Clinical Medicine, Department of Internal Medicine, University of Oulu, Oulu, Finland; 3 Medical Research Center Oulu, Oulu University Hospital and University of Oulu, Oulu, Finland; 4 Faculty of Biochemistry and Molecular Medicine, Oulu Center for Cell-Matrix Research, University of Oulu, Oulu, Finland; 5 Department of Experimental and Clinical Medicine, Marche Polytechnic University, Ancona, Italy; 6 Institute of Biomedicine, Department of Physiology, University of Oulu, Oulu, Finland; 7 Department of Epidemiology and Biostatistics, MRC Health Protection Agency (HPA) Centre for Environment and Health, School of Public Health, Imperial College, London, United Kingdom; 8 Center for Life Course Epidemiology, Faculty of Medicine, University of Oulu, Oulu, Finland; 9 Unit of Primary Care, Oulu University Hospital, Oulu, Finland

## Abstract

Common variants of human fat mass- and obesity-associated gene *Fto* have been linked with higher body mass index, but the biological explanation for the link has remained obscure. Recent findings suggest that these variants affect the homeobox protein IRX3. Here we report that FTO has a role in white adipose tissue which modifies its response to high-fat feeding. Wild type and *Fto*-deficient mice were exposed to standard or high-fat diet for 16 weeks after which metabolism, behavior and white adipose tissue morphology were analyzed together with adipokine levels and relative expression of genes regulating white adipose tissue adipogenesis and *Irx3*. Our results indicate that *Fto* deficiency increases the expression of genes related to adipogenesis preventing adipocytes from becoming hypertrophic after high-fat diet. In addition, we report a novel finding of increased *Irx3* expression in *Fto*-deficient mice after high-fat feeding indicating a complex link between FTO, IRX3 and fat metabolism.

Genetic factors have been estimated to account for 40–70% of variations in body weight and composition^1^. Several independent studies have established a constant association between genetic variants in the fat mass- and obesity-associated gene (*Fto*) and higher body mass index in humans^2^,^3^,^4^,^5^. However, we have yet to underpin the molecular and pathophysiological mechanisms through which FTO might impact on weight gain. *Fto* mRNA is abundant in the cerebral tissues including the hypothalamic centers, pointing to a role for FTO in the control of appetite and energy balance^6^. However, a hypothalamus-specific *Fto* depletion in adult mice induced only a mild reduction in body weight indicating that FTO possesses additional effects in peripheral tissues which still have to be identified^7^. Yet, very little attention has been given to the characterization of FTO in fat tissue. Only recently, Tews and colleagues reported that FTO could play a role in the “browning process” of white adipose tissue, possibly controlling the ratio between white and brown adipocytes in white adipose depots also referred as beige adipocytes^8^. Their findings support a role for FTO in inhibiting uncoupling protein 1 (*Ucp1*) expression, which is a characteristic of both brown and beige adipocytes. The underlying molecular process linking the *Fto* gene with the regulation of adipocyte cell lineage will warrant further analysis, especially since a recent finding suggests a non-negligible role for brown adipose tissues in adult human metabolic response^9^. Nevertheless, white fat remains the most abundant type of fat depot in humans and research focusing on the role of FTO in the metabolism of white adipocytes is required. This can be achieved in mice by studying epididymal white adipose tissue (WAT) where the *in vivo* expression of *Ucp1* is absent under physiological conditions^10^. WAT does indeed play a key role in the response to diet through the storage of excess energy into lipid droplets. Several transcription factors, including bone morphogenetic proteins 2 and 4 (BMP2 and 4), CCAAT/enhancer binding proteins α, β, γ and δ (C/EBPα, C/EBPβ, C/EBPγ and C/EBPδ) and peroxisome proliferator activated receptor γ (PPARγ), have been shown to regulate the differentiation of adipocytes from the precursor cells (reviewed in Ref. 11). During adipogenesis, the white adipocyte finally acquires its functional characteristics allowing the storage of triglycerides and the control of energy balance via production of adipokines such as adiponectin and leptin. These factors can play a role both in adipocyte differentiation and in the metabolic adaptation.

Previous studies have revealed controversial effects of *Fto* mRNA expression in human adipose tissue. *Fto* expression was decreased in obesity depending on the adipose tissue depot^12^,^13^, whereas Samaras *et al*. found no difference in the *Fto* expression between obese and control individuals^14^. These incompatible results suggest a role for FTO in adipose tissue and demand further investigation. In addition, a recent finding suggested that part of the effect attributed to *Fto* polymorphism in humans could be due to long-distance gene-gene interaction and alteration upon the expression of the iroquois homeobox gene 3 (*Irx3*)^15^. Interestingly, the *Irx3* gene is up-regulated together with weight loss in human adipocytes^16^ suggesting a role for IRX3 in adipocyte metabolism. As to whether *Irx3* gene expression can be modified in response to invalidation of the *Fto* gene in mouse is wholly unknown.

The focus of the current study is the effects of FTO in the white adipocyte differentiation and function. Mouse epididymal WAT has been referred as “pure” white adipose tissue as it under physiological conditions is not prone to the browning process^10^. This depot has no clear human counterpart as retroperitoneal or inguinal WAT would have, but those are however more susceptible to the browning process and thus not an optimal choice for analysis of white adipocytes *in vivo*. In the current study, we investigated the effect of *Fto* depletion on genes related to adipogenesis, WAT function as well as *Irx3* expression. Evidence from human epidemiology and animal models has pointed to a role for FTO in food intake but also in food choice and adaptation to diet (see Ref. 17 for review). While it is necessary to characterize the role of FTO in the normal physiological state, with a control diet (CD), it is also important to characterize its function in response to the stimulation of WAT during consumption of a high-fat diet (HFD). The present study focuses on the direct influence of FTO in WAT biology in the * Fto-knockout (Fto*-KO) mouse model produced using embryonic stem cells with a “knock-in-first” targeting construct, generated by the European Conditional Mouse Mutagenesis Program (EUCOMM). The role of FTO in WAT biology and adipogenesis *in vivo* is investigated, together with how the dietary environment can provide new insights into the ways that FTO can control body weight regulation.

## Results

### Generation and characterization of *Fto*-KO mouse model

**Figure 1:**
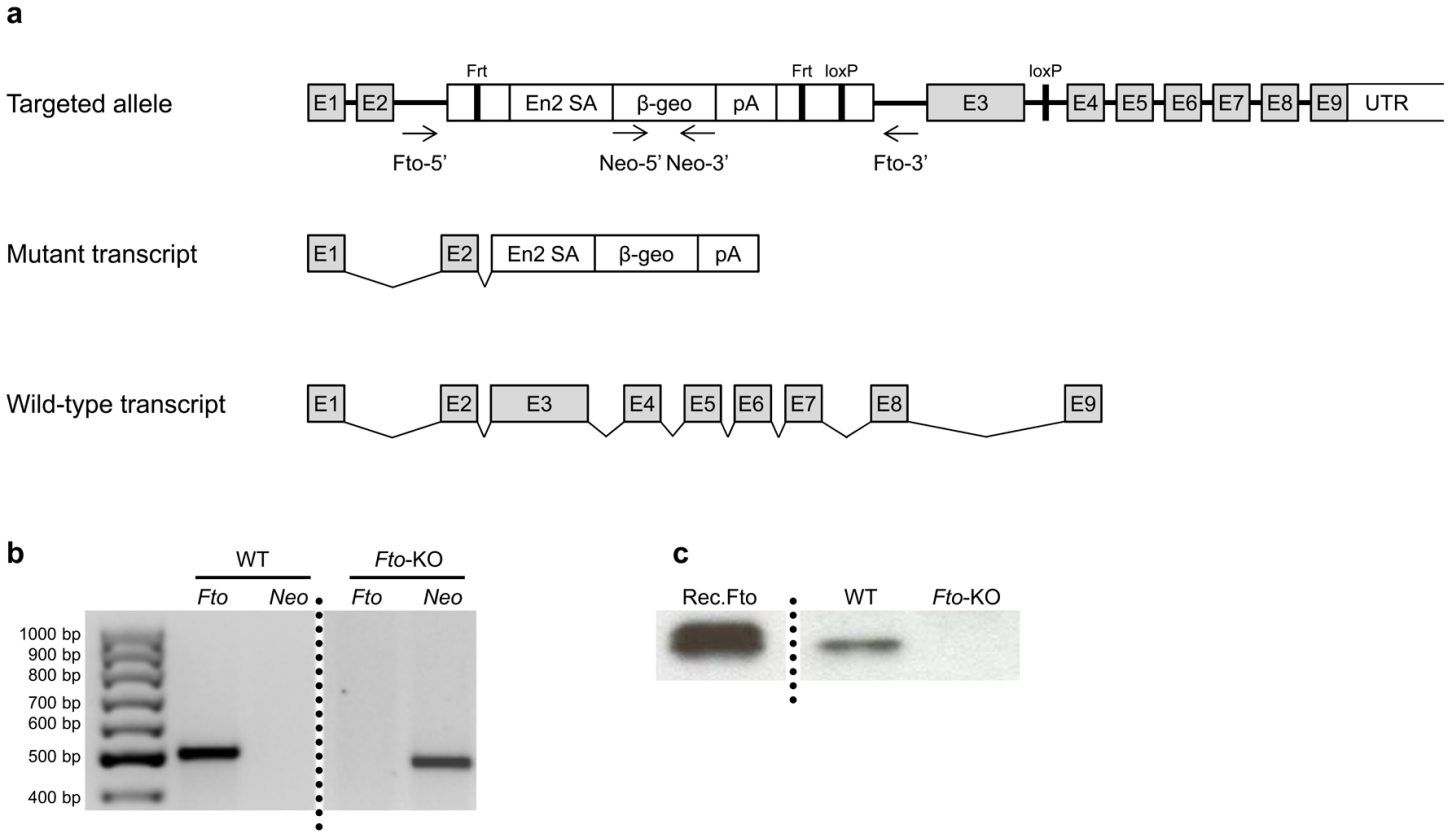
Disruption of the mouse *Fto* gene. *WT* wild type; *Fto-KO* *Fto*-knockout. (a) An embryonic stem cell line containing a ‘knock-in-first’ construct targeting the second intron of mouse *Fto* gene, disrupting the endogenous transcription and leading to production of truncated mRNA, was used to generate *Fto*-KO mouse line. *Arrows* represent the primer binding sites used in the genotyping; *E1*–*E9* exons 1–9; *En2SA* En2 splice acceptor; *β-geo* fusion of β galactosidase and neomycin resistance genes; *pA* polyadenylation signal; *UTR* untranslated region; *Frt* flippase recognition target site; *loxP* locus of crossover in P1 site. (b) Genotyping of WT and *Fto*-KO mice by PCR. A 500-bp fragment was produced from the WT allele with *Fto*-specific primers which lack the *Fto*-KO allele due to the construct. Primers targeting the neomycin resistance gene (*Neo*) produced a 475-bp fragment from the *Fto*-KO construct which was not present in the WT allele. (c) Absence of FTO protein in *Fto*-KO mice was confirmed with Western blot by using an antibody against mouse FTO and donkey anti-rat IgG as a secondary antibody. Recombinant FTO (*Rec. Fto*) was used as a positive control (60 kDa) which was run in the same gel and under same experimental conditions as the samples. Dotted lines indicate the cropping lines.

The correct targeting of the *Fto* gene was verified by PCR (Fig. 1a and b) and complete depletion of the FTO protein by Western blotting (Fig. 1c). Genotyping of pups obtained from heterozygous matings revealed that all three genotypes were born in the normal Mendelian ratios.

**Figure 2:**
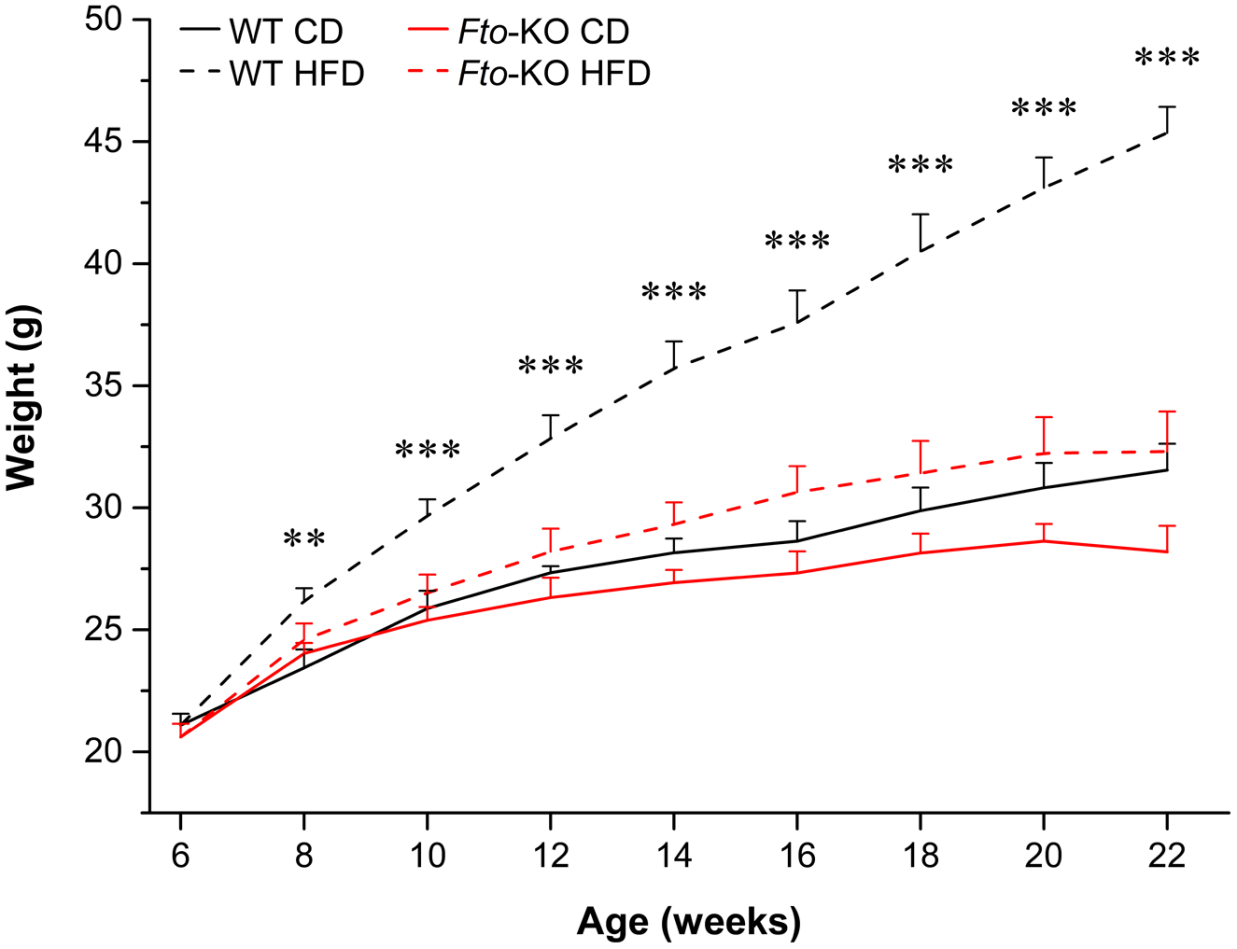
Weight development of mice during the dietary experiment. *WT* wild type; *Fto-KO* *Fto*-knockout; *CD* control diet; *HFD* high-fat diet. Results are shown as mean ± SEM (n = 5–9 per group) Two-way ANOVA followed by simple main effects analysis with Bonferroni correction because of the significant interaction effect in the repeated measures ANOVA, **p < 0.01, ***p < 0.001 compared to WT mice fed HFD.

The final weights of each dietary group were on average 31.5 ± 1.1 g and 28.2 ± 1.1 g, respectively, for wild type (WT) and *Fto*-KO mice fed a CD, and 45.4 ± 1.1 g and 32.3 ± 1.6 g, respectively, for WT and *Fto*-KO fed a HFD (Fig. 2). Critically, the body weights of the WT and *Fto*-KO animals were similar at the age of six weeks, however the WT mice fed HFD gained more weight from the age of eight weeks onwards in comparison to their *Fto*-KO counterparts. Weights and metabolic parameters are summarized in Table 1. Interestingly, despite marked differences in body weight between the WT and *Fto*-KO mice fed HFD, we did not observe any difference in either water or food intake or any other measures of energy expenditure. The parameters of energy balance were altered in response to HFD, but the genotype exerted no supplementary effect.

### Adipocyte morphology and genes associated with adipogenesis and white adipose tissue function after HFD

**Figure 3:**
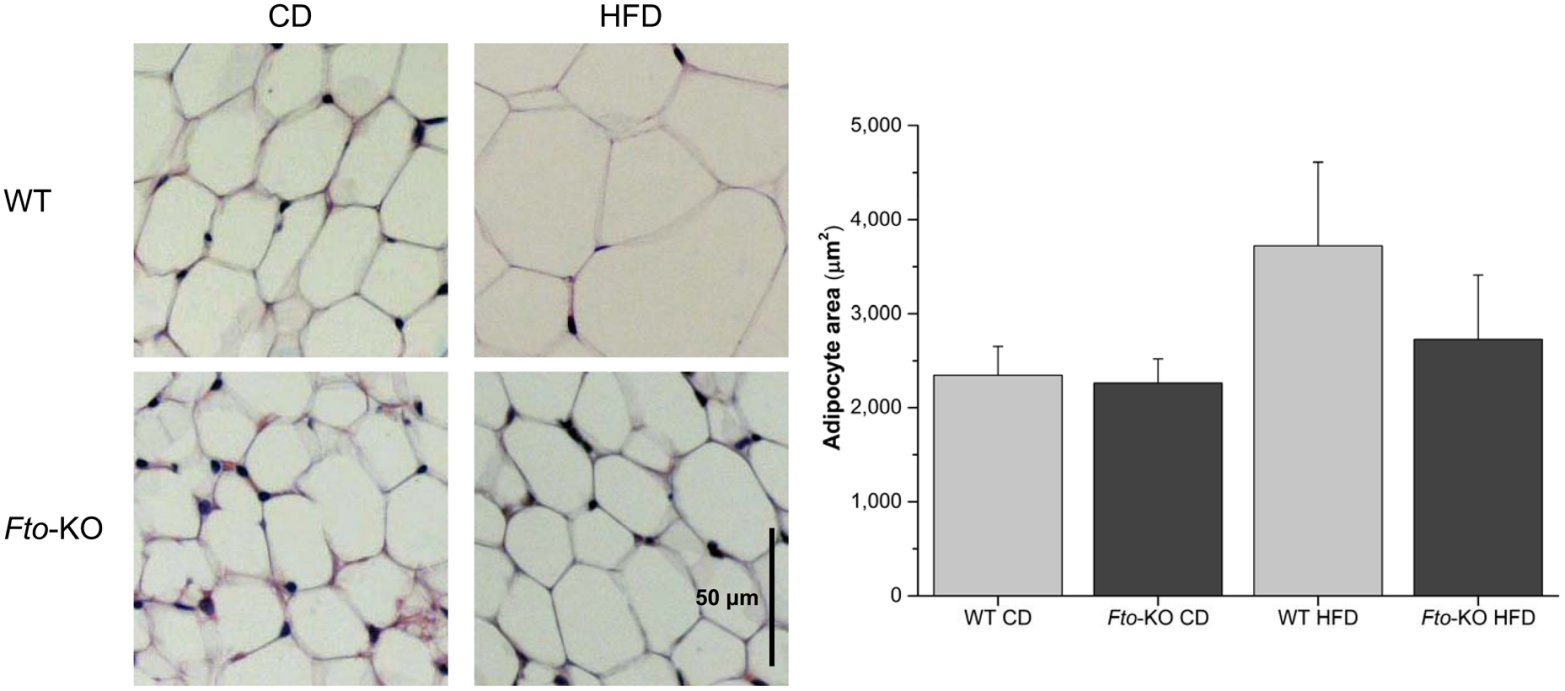
Hematoxylin-eosin staining of mouse white adipose tissue. *WT* wild type; *Fto-KO* *Fto*-knockout; *CD* control diet; *HFD* high-fat diet. Scale bar is 50 μm and adipocyte area is shown as mean ± SEM (n = 3 mice per group, 300 cells per mouse).

**Figure 4:**
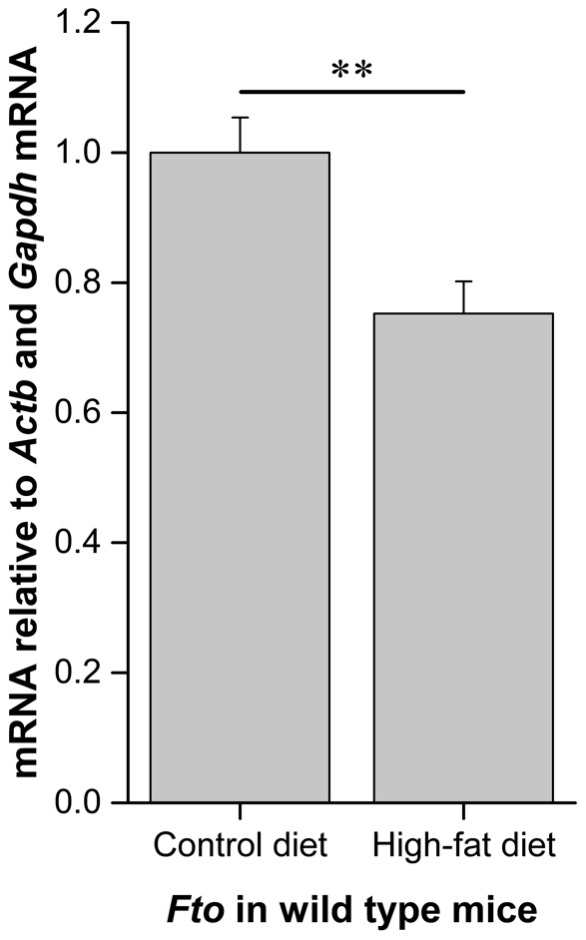
Relative expression of *Fto* in white adipose tissue. Amount of *Fto* mRNA was normalized using *Actb* and *Gapdh* as reference genes. Results are shown as mean ± SEM (n = 9 per group). Student's t-test, **p < 0.01.

**Figure 5:**
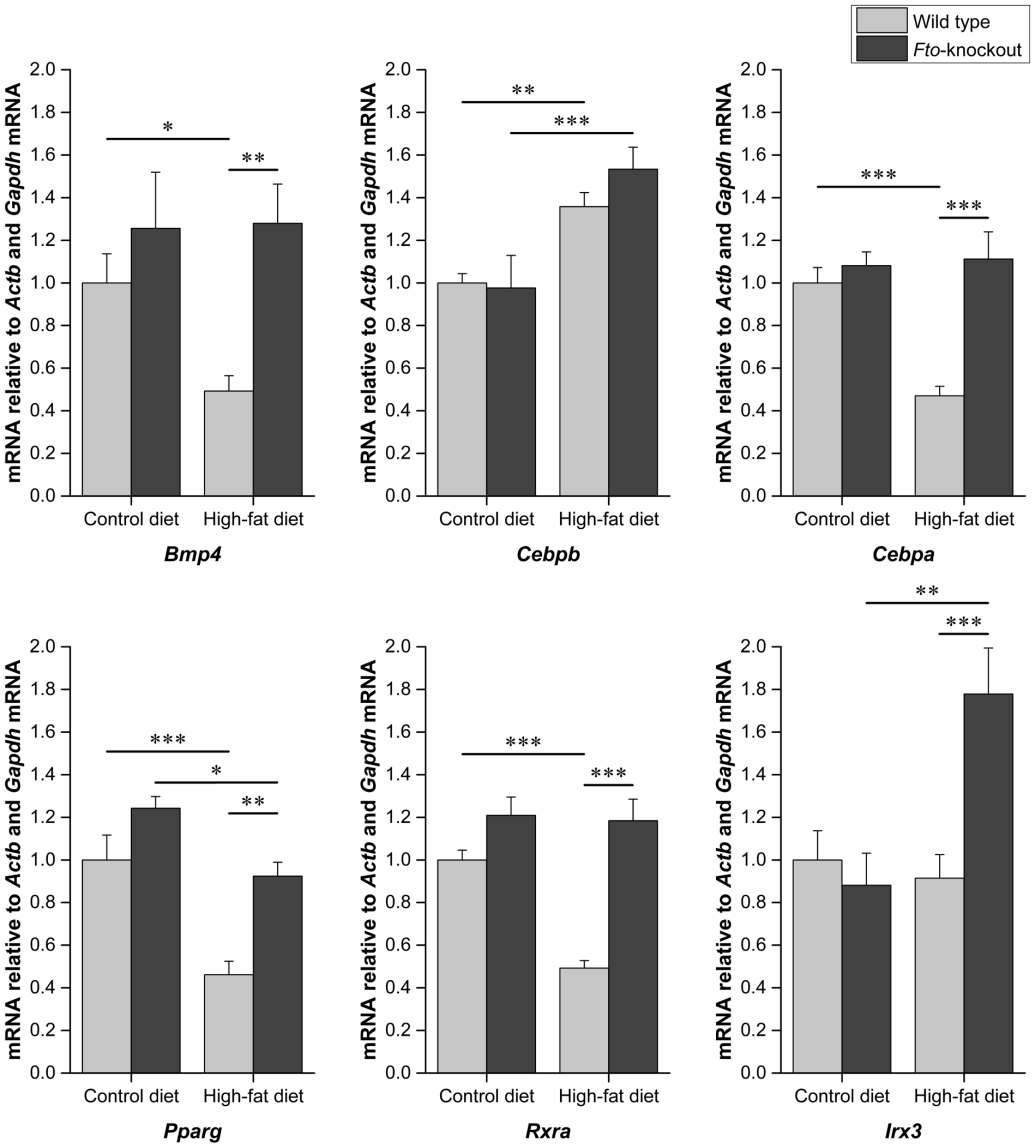
Relative expression of genes related to adipogenesis in white adipose tissue. Amount of mRNA was normalized using *Actb* and *Gapdh* as reference genes. Results are shown as mean ± SEM (n = 5–9 per group). Two-way ANOVA followed by simple main effects analysis with Bonferroni correction because of the significant interaction effect, *p < 0.05, **p < 0.01, ***p < 0.001.

**Figure 6:**
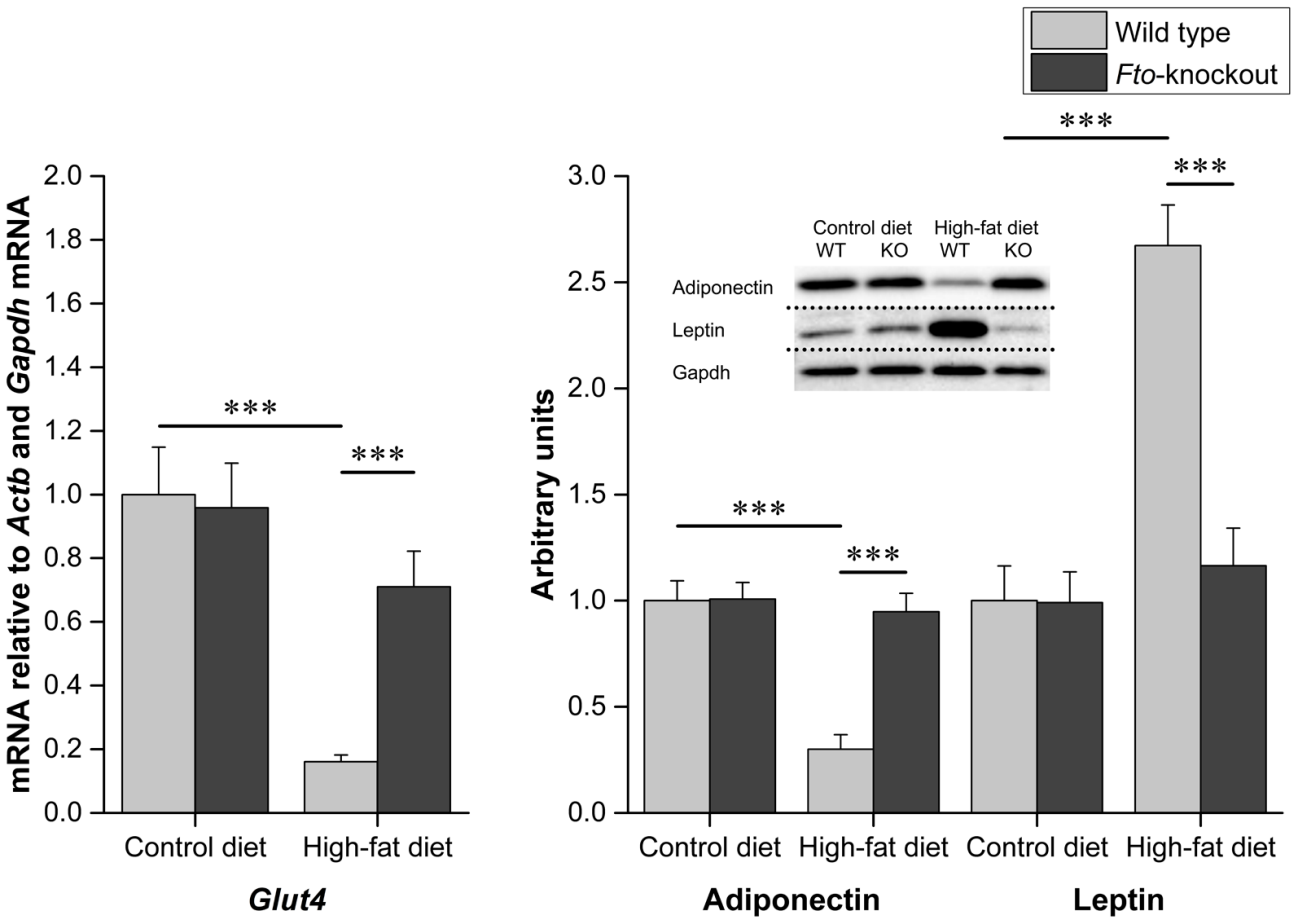
Protein level of adipokines and relative expression of *Glut4* in the white adipose tissue. Band intensities were analyzed from membranes relative to protein amount loaded to the gel. All samples were run under same experimental conditions and dotted lines indicate the cropping lines. Amount of *Glut4* mRNA was normalized using *Actb* and *Gapdh* as reference genes. Results are shown as mean ± SEM (n = 5–9 per group in gene expression analysis and n = 6 per group in protein analysis). Two-way ANOVA followed by simple main effects analysis with Bonferroni correction because of the significant interaction effect, *p < 0.05, **p < 0.01, ***p < 0.001.

The mean adipocyte area in WAT is shown in the Figure 3. As expected, WT mice fed HFD show enlargement of adipocytes. However, feeding HFD in *Fto*-KO mice had no effect on the adipocyte size. *Ucp1* gene expression was not detected in the epididymal WAT, supporting the previously reported characteristic of white adipocytes^10^. The relative expression of *Fto* in WT mice is shown in Figure 4. *Fto* was 1.3-fold lower in the WAT of WT mice fed HFD (*p* < 0.01). Furthermore, also relative expression of genes related to adipogenesis and adipose tissue function, *Bmp4*, *Cebpb*, *Cebpa*, *Pparg*, *Rxra* and *Glut4*, was studied in the epididymal WAT of WT and *Fto*-KO mice. Expression of genes related to adipogenesis and development are illustrated in Figure 5. When mice were fed CD, the *Bmp4* expression was 1.3-fold higher in *Fto*-KO mice compared to WT. HFD lowered the *Bmp4* expression by 2-fold in WT whereas the *Bmp4* level was unaltered in *Fto*-KO mice after HFD. However, there was no difference in the expression of *Cebpb* between WT and *Fto*-KO mice while the consumption of HFD was associated with a 1.4- and 1.5-fold increased *Cebpb* expression in WT and *Fto*-KO mice, respectively. The relative expression of *Cebpa* was unaltered between the genotypes when fed CD. However, HFD introduced a 2.1-fold decrease in *Cebpa* expression in WT mice, which was not seen in *Fto*-KO mice. When fed CD, the relative expression of *Pparg* was 1.2-fold higher in *Fto*-KO when compared to WT mice. In both, WT and *Fto*-KO mice, the expression level of *Pparg* was decreased due to HFD, 2.2-fold in WT but only 1.3-fold in *Fto*-KO mice. A similar trend was also seen in the expression of *Rxra*, the heterodimeric counterpart of PPARY. *Irx3* expression was unaltered in mice fed CD while HFD led to 1.8-fold increase of *Irx3* in *Fto*-KO mice. Such an effect was not observed in the WT mice (Fig. 5). The *Glut4* expression of *Fto*-KO mice was at the same level as in WT mice, and HFD lowered the expression by 6.2-fold in WT mice but only by 1.4-fold in *Fto*-KO (Fig. 6).

### Effect of *Fto* depletion on adiponectin and leptin synthesis

The protein levels of adiponectin and leptin in the epididymal WAT of *Fto*-KO mice were studied with Western blot. There was no difference in adiponectin and leptin levels between the mice when fed CD (Fig. 6). However, adiponectin was reduced by 3.3-fold in WT when fed HFD, which was not seen in the *Fto*-KO mice. Leptin on the other hand was 2.7-fold higher in WT mice after HFD, which was not seen in the *Fto*-KO mice.

## Discussion

The current study is the first to report the response of an *Fto*-KO mouse model to high-fat feeding intending to identify the role of FTO in white fat cells. The present report provides new evidence for a role of *Fto* in the expression of genes related to adipogenesis, adipocyte functions and adipokine production. The exposure to HFD emphasizes the effect of *Fto* depletion, suggesting that FTO has a role in adipocyte hypertrophy. The strength of the current study is that the effects of HFD are investigated in relation to CD. In this analysis, we see that the response of *Fto*-deficient adipose tissue to increased nutritional fat is different than that of wild type adipose tissue. Whether the difference rise from altered adipogenesis *per se* or other developmental change cannot be estimated from the data represented here, but it unquestionably demand further studies.

We observed that the adipocyte size in *Fto*-KO mice was smaller when compared to WT after HFD. This might be fundamental to understand the functionality of FTO in the etiology of obesity and related diseases. In fact, the capacity of white adipocytes to become hypertrophic in response to obesity is a key factor in the pathogenicity of excessive weight gain^18^. The size of the white adipocyte is highly correlated with its capacity to become insulin resistant^19^ and also associated with the pro-inflammatory activity of the white adipose tissue as a result of various mechanisms including the endoplasmic reticulum stress response^20^. In the current study, the fat and lean mass was not quantified separately hence the number of adipocytes cannot be directly examined. Previous reports show controversial effects of *Fto*-KO on fat mass. Generally, the fat mass is either reduced^7^,^21^,^22^ or unaltered^23^ when compared with WT which was also seen after HFD. In addition, the International Mouse Phenotyping Consortium database (www.mousephenotype.org) show unaltered fat mass for the mouse model closest to one presented here (Fto^tm1a(EUCOMM)Wtsi^)^24^. Thus, in light of previous studies we may postulate that the fat mass is at least not increased in the current *Fto*-KO model. As a summary, unaltered body and fat mass, unaltered adipocyte area of *Fto*-KO mice after CD and the lack of hyperthropy after HFD, we may conclude that the number of adipocytes is unaltered in the *Fto*-KO mice reported here. This indicates that the capacity of *Fto*-KO adipose tissue to store the nutritional fat is altered when the standard conditions are challenged by HFD.

The current *Fto*-KO mouse model differs from previous models^7^,^21^,^23^ as this “knock-in-first” model terminates the mRNA production inside second intron of *Fto* gene without deleting any part of the genome. This model thus allows the regulation of other genes to occur via non-coding regions of *Fto*. The main phenotypic characteristic of the present model in comparison to previously published *Fto*-KO mice lies in the fact that the weight gain of the KO animals appeared similar to the one of WT animals when fed CD. This more subtle alteration in body weight is closer to another mouse model without deletion of the genome generated by point-mutation inside the coding region, in which the *Fto*-KO mice possessed weight reduction from 12 weeks of age onwards when compared to WT mice^22^. Concurrently, no increased postnatal mortality was observed with the present *Fto*-KO mice which is similar to the model developed by Church and colleagues^22^. A recent study suggested that the association between human SNPs in the *Fto* and higher BMI could in fact be due to long-range regulatory alteration of the homeobox gene *Irx3*^15^. In addition, *Irx3*-deficient mice exhibited reduced body weight from an early age compared to WT mice^15^. IRX family members are expressed ubiquitously during development and are important in the regionalization of cell differentiation (reviewed in Ref. 25). The current study is the first to report the effect of FTO on *Irx3* expression. Interestingly, *Irx3* was not altered in mice fed CD but was significantly increased due to HFD in *Fto*-KO mice only. In light with previous findings, our data suggests that the *Irx3* overexpression could explain part of the effects of the invalidation of FTO. However, we also observed that while *Fto* gene expression was down-regulated in WT mice fed HFD, there was no change in the *Irx3* expression. This indicates a complex molecular pathways linking FTO, IRX3 and fat metabolism that warrant further investigation both in wild type and knockout models.

We did not observe difference in energy expenditure between the WT and *Fto*-KO mice, which is in line with the conclusion of McMurray *et al.*^7^. On the contrary, a previous study reported that the leanness of the *Fto*-deficient mice was a consequence of increased energy expenditure^21^. This discrepancy may result from the fact that in the present study, the effects of different diets were analyzed after a relatively long period. After 16 weeks of dietary treatment, the mice of either genetic background did not exhibit any specific changes related to energy intake, physical activity or heat production. Importantly, the data from human studies suggest an age dependent effect of *Fto* polymorphism. For instance, the current knowledge based on cross-sectional and longitudinal analysis has pointed to a stronger association of *Fto* polymorphism with BMI for the age of adiposity rebound^26^. This association remains consistent in early adulthood but tends to be lost in older age^27^. Thus, it is reasonable to hypothesize that the onset of the changes starts early in the development, and with older mice, compensatory effects may have diminished the differences. This issue has received very little attention by far and remains to be clarified elsewhere.

The results of the present study indicate that the white adipose tissue of *Fto*-KO mice responds differently when stimulated by HFD. Genes related to adipogenesis, such as *Bmp4*, *Cebpa*, *Pparg* and *Rxra*, are expressed higher in the WAT of *Fto*-KO mice and HFD feeding does not cause a similar reduction as seen in WT mice. The precise mechanism through which FTO regulates energy metabolism and the response to the diet is unknown. The *Fto* gene is known to encode a 2-oxoglutarate-dependent RNA demethylase and we have yet to establish its specific role in body weight regulation^28^,^29^. FTO has been speculated to be involved in the sensing of cellular energy levels and *Fto* expression is found to be associated with cellular adenosine triphosphate (ATP) concentration^30^. Recently, *in vitro* studies demonstrated a potential role for FTO in the nutrient sensing process, particularly for amino acids^31^. The authors tested the effects of FTO on the mammalian target of rapamycin (mTOR) signaling pathway, which is one of the key regulators of mRNA translation and cell growth (reviewed in Ref. 32). Mouse embryonic fibroblasts (MEFs) from *Fto*-deficient mice displayed decreased mTOR complex 1 (mTORC1) signaling and mRNA translation in conjunction with increased autophagy^31^. Another study revealed that total inhibition of mTOR signaling prevented the 3T3-L1 preadipocytes from differentiating into mature adipocytes, while a partial knockdown of mTOR may in fact enhance adipogenesis, as reflected by the increased expression of adipocyte markers such as *Pparg* and *Cebpa*^33^. Thus, mTOR has been postulated to exert a homeostatic role so that the level of mTOR signaling acts to either promote or suppress adipogenesis. Altered mTOR signaling may have contributed to the changes seen in WAT of our *Fto*-KO mice since, in addition to altered adipose tissue morphology, the expression of *Bmp4*, *Cebpa*, *Pparg* and *Rxra* was significantly increased in the WAT of *Fto*-KO mice after HFD. Interestingly, our results indicate that *Fto* deficiency does not have an effect on the expression levels of *Cebpb*, an adipogenic transcription factor activating PPARγ and C/EBPα, suggesting a C/EBPβ-independent route for FTO action. *Cebpb* mRNA levels were higher due to HFD in both genotypes which is in line with a previous study^34^.

Adiponectin and leptin concentrations in serum have been shown to vary in relation to the amount of adipose tissue^35^,^36^. In addition, the serum level of adiponectin was increased and that of leptin decreased in the previously published report of *Fto*-KO mice^21^. According to our results, adiponectin was not reduced in the epididymal WAT of *Fto*-KO mice due to high-fat diet, as was seen in the WT mice. On the contrary, leptin was increased in the WT mice due to high-fat diet while it remained at the same level in the *Fto*-KO mice. This is in agreement with the previous studies indicating that leptin levels are higher in obesity^37^.

In conclusion, our study reveals that the characteristics of white adipose tissue are altered in *Fto*-deficient mice. These mice are leaner than WT mice when fed a HFD. In addition, we found that *Fto* was down-regulated due to HFD in WT mice, an effect also associated with increased adipocyte size. This appears to be opposite to the situation in *Fto*-KO adipocytes, which are smaller due to the absence of *Fto* with increased *Irx3* expression. The discrepancy may indicate that the differences between *Fto*-KO and WT adipocytes originate from an earlier developmental phase which contributes to the altered metabolic performance. Our study shows that the expression of genes regulating adipogenesis is affected in *Fto*-deficient mice. This results in smaller adipocytes as well as altered adipokine production and alters the response of adipose tissue to high-fat diet.

## Methods

### Generation of *Fto*-KO mice

C57BL/6N embryonic stem cells targeted with the “knock-in-first” construct were obtained from the EUCOMM (www.eucomm.org). To generate the *Fto*-KO mouse line, the clone EPD0103_5_G10 carrying a targeted *Fto* allele was used in blastocyst injections in the transgenic core facility of Biocenter Oulu. The analyses described here were carried out in mice which had been backcrossed to the C57BL/6JOlaHsd background for up to three generations. *Fto*-KO mice refer to the mice containing the whole KO-cassette which, due to polyadenylation signal, causes termination of *Fto* gene transcription inside the second intron without deleting any sequence of the gene. The genotype was confirmed by PCR using primers specific for Fto (forw 5′-TTACTGCATTCTCTTCCAGAGAGG and rev 5′-ACGCCCAGACACTCTCCAGTGAGC) and Neo (forw 5′-TGTCATCTCACCTTGCTCCTG and rev 5′-TCAAGAAGGCGATAGAAGGCG). Wild type (WT) controls were C57BL/6JOlaHsd mice obtained from the Laboratory Animal Center, University of Oulu. All animal experiments were approved by the University of Oulu Animal Ethics Committee and the Southern Finland Regional State Administrative Agency (license numbers ESLH-2008-06715/Ym-23 and ESAVI/4464/04.10.03/2011). The animal care and procedures for the animal experiments followed the national Finnish legislation, the European Convention for the protection of vertebrate animals used for experimental and other scientific purposes (ETS 123) and EU Directive 2010/63/EU.

### Dietary treatment

All mice used in the experiments were housed in the Laboratory Animal Center, University of Oulu, at 21 +/− 2°C on a 12/12-hour dark-light cycle and had *ad libitum* access to water and food. Analyses were performed on male mice only. Six-week-old animals were randomly allocated into four groups in order to test the effects of both genotype and dietary treatment: WT mice fed a control diet (WT CD, n = 9), *Fto*-KO mice fed a control diet (*Fto*-KO CD, n = 5), WT mice fed a high-fat diet (WT HFD, n = 9) and *Fto*-KO mice fed a high-fat diet (*Fto*-KO HFD, n = 8). The control diet (CD) contained 10 kJ-% fat, 20 kJ-% protein and 70 kJ-% carbohydrate (total energy 16.1 kJ g-1) while the high-fat diet (HFD) had 45 kJ-% fat, 20 kJ-% protein and 35 kJ-% carbohydrate (total energy 19.8 kJ g-1) (D12450B and D12451, Research Diets, New Brunswick, NJ, USA). The mice were weighed every two weeks and sacrificed by carbon dioxide inhalation and cervical dislocation after 16 weeks of dietary treatment. Epididymal WAT samples were snap-frozen in liquid nitrogen and stored at −70°C until subsequent RNA and protein isolations.

### Metabolic and physical performance

After 16 weeks of dietary treatment, metabolic performance, physical activity as well as drinking and feeding behavior were analyzed in a home cage-based monitoring system for laboratory animals (LabMaster TSE System, Bad Homburg, Germany) as described previously^38^. During the experiment, the mice were housed in separate cages and, after a one week acclimatization period, O_2_ consumption, CO_2_ production, respiratory exchange rate and heat production were determined with an indirect calorimetric system. Physical activity comprising ambulatory and fine movements was detected by infrared light-beam sensors surrounding the cage. Water and food consumption were measured by sensors attached to the cage lids. Each parameter was measured once every 30 minutes during a total period of 95 hours.

### Light microscopy

After dissection, samples of the adipose tissue depots were fixed in 4% paraformaldehyde in 0.1 M phosphate buffer, pH 7.4 overnight at 4°C. After rinsing in 0.1 M phosphate buffer, specimens were dehydrated in ethanol, cleared in xylene and embedded in paraffin. Serial paraffin sections with a thickness of 3 μm were obtained from each specimen. The sections were stained with hematoxylin and eosin to assess morphology. Tissue sections were imaged with a Nikon Eclipse E800 light microscope using a 40× objective and the digital images were captured with a Nikon DXM 1200 camera. The mean area of adipocytes was calculated from three epididymal WAT samples per group and 300 cells per mouse using Nikon LUCIA IMAGE version 4.61 (Laboratory Imaging, Prague, Czech Republic).

### Protein extraction and Western blotting

Proteins were extracted from the epididymal WAT of six mice per group with the standard protocol. Briefly, 2.5 volumes of lysis buffer (20 mM Tris-HCl pH 7.5, 150 mM NaCl, 1% Triton-X, 2 mM EDTA, 10 × Protease Inhibitor Cocktail (Sigma-Aldrich, St-Louis, MO, USA)) were added to 100 mg of frozen tissue and homogenized with Tissue-Lyser LT (Qiagen, Hilden, Germany) in +4°C for 2 min at 35 Hz, then centrifuged in +4°C for 10 min at 18,000 × G. Equal amounts of protein were loaded to 4/20% SDS-PAGE gel and blotted to nitrocellulose or polyvinylidene fluoride membrane. Antibody against mouse FTO (AG-20A-0088, AdipoGen International, San Diego, CA, USA) was used with donkey anti-rat IgG (NBP1-73749, Novus Biologicals, Littleton, CO, USA) as a secondary antibody. Antibody against adiponectin (PA1-054) was obtained from Affinity Bioreagents (Golden, USA) and leptin antibody (500-P68) was from PeproTech (Rocky Hill, NJ, USA). Glyceraldehyde-3-phosphate dehydrogenase (GAPDH) was used as loading control with the antibody from Santa Cruz Biotechnology (sc-25778, Dallas, TX, USA). For adiponectin, leptin and GAPDH, the secondary antibody was donkey anti-rabbit IgG (NA934, GE Healthcare, UK). Pierce ECL Western Blotting Substrate (Thermo Scientific, Rockford, IL, USA) was used for the detection of the immunocomplexes on the membrane. Band intensities of adiponectin and leptin relative to the protein amount loaded to the gel were calculated using Fiji image analysis software^39^.

### RNA isolation and cDNA synthesis

After 16 weeks on either CD or HFD, WT and *Fto*-KO mice were sacrificed and 50–100 mg of epididymal WAT was collected. Total RNA was isolated with RNeasy Mini Kit after homogenization with QIAzol Lysis Reagent and TissueLyser LT according to the manufacturer's instructions (Qiagen, Hilden, Germany). Concentration and quality of the RNA were estimated with NanoDrop ND-1000 UV-Vis Spectrophotometer (NanoDrop Technologies, Wilmington, DE, USA). The genomic DNA was removed from the RNA sample with DNAase treatment and cDNA was synthetized from 500 ng of total RNA using RevertAid™ First Strand cDNA Synthesis Kit (Fermentas, Sankt Leon-Rot, Germany).

### Quantitative real-time reverse-transcriptase polymerase chain reaction (RT-qPCR)

Relative expression of *Bmp4*, *Cebpb*, *Cebpa*, *Pparg*, *Rxra*, *Irx3*, glucose transporter 4 (*Glut4*), and *Ucp1* from mouse epididymal WAT were studied. As reference genes, β-actin (*Actb*, for ‘5- TGGATCGGTGGCTCCATCCTGG and rev 5′- CGCAGCTCAGTAACAGTCCGCCTA) and *Gapdh* (for 5′- CCAATGTGTCCGTCGTGGATCT and rev 5′- GTTGAAGTCGCAGGAGACAACC^40^) were used. All primers were obtained from Sigma-Aldrich (St. Louis, MO, USA). The reactions were performed in a 25 μL reaction mixture containing 12.5 μL iQ™SYBRGreen Supermix (Bio-Rad Laboratories, Hercules, CA, USA), 0.5 μL Forward primer (20 μM), 0.5 μl Reverse primer (20 μM), 6.5 μL sterile water and 5 μL of the cDNA sample dilution. The protocol proceeded as follows: 3 min 95°C; 40 cycles at 10 sec 95°C, 10 sec annealing temperature, 10 sec 72°C; 2 min 72°C. Melting curve analysis was included at the end of every run: 55°C to 95°C at 0.5°C intervals, 10 sec at each temperature. The annealing temperatures as well as sequences for all primer pairs used are shown in Supplementary Table 1. Each sample was analyzed in duplicate and interrun calibrator and no-template control were used in all the plates. For *Ucp1* expression studies, cDNA from brown adipose tissue was used as a positive control. Ct values were collected using iCycler thermal cycler and iQ5 Real-Time Detection System (Bio-Rad Laboratories, Hercules, CA, USA). Relative expression of each gene was calculated using the 2^−*ΔΔCt*^ method^41^.

### Statistics

Distributions of all variables were tested with the Shapiro-Wilk test and equalities of variance with Levene's test. For skewed distribution and/or unequal variance, logarithmic transformation was applied. Difference in weight development between the groups studied was analyzed with two-way ANOVA with simple main effects analysis at individual time points since there was a significant interaction effect (Wilks' Lambda *p* < 0.001) present with the repeated measures ANOVA. The characteristics of the study groups and differences in relative expression of genes as well as protein levels were analyzed with two-way ANOVA followed by simple main effects analysis with Bonferroni correction if there was a significant interaction effect. Difference in relative expression of *Fto* in WT mice after consumption of CD or HFD was analyzed with Student's t-test. *P* values lower than 0.05 were considered statistically significant with the results being expressed as mean and SEM unless indicated otherwise. Statistical analyses were conducted using IBM SPSS Statistics software package version 20.0 (IBM, Armonk, NY, USA).

## Author Contributions

J.R., S.S. and T.S. wrote the main manuscript text. J.R. and T.J.H. conducted the statistical analyses. R.S. and T.S. produced the *Fto*-deficient mice. J.R., E.M., F.C., K.A.M., M.K. and T.S. conducted the laboratory work. K.-H.H., M.-R.J. and M.J.S. contributed to the reagent, material and analysis tools. All authors reviewed the manuscript.

## Supplementary Material

Supplementary Information Supplementary Information

## Figures and Tables

**Table 1:** Phenotype of mice after 16 weeks' consumption of control or high-fat diet. *CD* control diet; *HFD* high-fat diet. Results are shown as mean ± SEM and n = 5–9 per group. Metabolic and physical parameters are indicated as mean per day ± SEM. *P* values are obtained with two-way ANOVA. Simple main effects analysis with Bonferroni correction was conducted if there was a significant interaction effect (genotype × diet), after which: ^a^*p* < 0.001 compared to WT mice fed HFD

				*p* values		
		Wild type	*Fto*-knockout	Effect of genotype	Effect of diet	Genotype × diet
**Final weight**	**CD**	31.5 ± 1.09^a^	28.2 ± 1.06	<0.001	<0.001	0.001
(g)	**HFD**	45.4 ± 1.07	32.3 ± 1.64^a^			
**Water intake**	**CD**	3.74 ± 0.34	3.03 ± 0.26	0.35	<0.001	0.14
(mL day-1)	**HFD**	2.28 ± 0.26	2.45 ± 0.22			
**Energy intake**	**CD**	62.7 ± 2.8	67.0 ± 5.3	0.33	0.77	0.82
(kJ day-1)	**HFD**	59.6 ± 7.9	66.7 ± 3.8			
**Heat production**	**CD**	95.3 ± 2.1	96.3 ± 1.7	0.79	0.12	0.99
(kJ h-1 day-1)	**HFD**	100.8 ± 3.7	101.7 ± 3.7			
**Heat production per weight**	**CD**	3.30 ± 0.11	3.56 ± 0.22	0.40	<0.001	0.36
(kJ h-1 g-1 day-1)	**HFD**	2.64 ± 0.15	2.63 ± 0.11			
**Respiratory exchange rate**	**CD**	45.1 ± 0.71	45.1 ± 1.03	0.53	<0.001	0.48
(VCO_2_ VO_2_-1 day-1)	**HFD**	37.9 ± 0.62	38.8 ± 0.37			
**Ambulatory movement**	**CD**	8429 ± 1577	10098 ± 1190	0.79	0.01	0.24
(counts day-1)	**HFD**	6596 ± 593	5527 ± 951			
**Fine movement**	**CD**	6546 ± 1169	7829 ± 774	0.66	0.09	0.30
(counts day-1)	**HFD**	5927 ± 400	5395 ± 790			
**Total movement**	**CD**	17480 ± 1312	17927 ± 1948	0.90	<0.001	0.81
(counts day-1)	**HFD**	12523 ± 970	12386 ± 821			
