# Supplementary material for: Fat mass- and obesity-associated gene Fto affects the dietary response in mouse white adipose tissue
Source: Sci Rep. 2015 Mar 18;5:9233. doi: 10.1038/srep09233 (PMC4363842; doi:10.1038/srep09233)
Supplement: Supplementary Information [file srep09233-s1.pdf]

SUPPLEMENTARY INFORMATION

Fat mass- and obesity-associated gene *Fto* affects the dietary response in mouse white adipose tissue

Justiina Ronkainen[1,2,3], Tuija J. Huusko[1,2,3], Raija Soininen[1,4], Eleonora Mondini[5], Francesca Cinti[5], Kari A. Mäkelä[1,3,6], Miia Kovalainen[1,3,6], Karl-Heinz Herzig[1,3,6], Marjo-Riitta Järvelin[7,8,9], Sylvain Sebert[1,10], Markku J. Savolainen[1,2,3] & Tuire Salonurmi[1,2,3]

1 Biocenter Oulu, University of Oulu, Oulu, Finland; 2 Institute of Clinical Medicine, Department of Internal Medicine, University of Oulu, Oulu, Finland; 3 Medical Research Center Oulu, Oulu University Hospital and University of Oulu, Oulu, Finland; 4 Department of Medical Biochemistry and Molecular Biology, Oulu Center for Cell-Matrix Research, University of Oulu, Oulu, Finland; 5 Department of Experimental and Clinical Medicine, Marche Polytechnic University, Ancona, Italy; 6 Institute of Biomedicine, Department of Physiology, University of Oulu, Oulu, Finland; 7 Department of Epidemiology and Biostatistics, MRC Health Protection Agency (HPA) Centre for Environment and Health, School of Public Health, Imperial College, London, United Kingdom; 8 Unit of Primary Care, Oulu University Hospital, Oulu, Finland; 9 Department of Children and Young People and Families, National Institute for Health and Welfare, Oulu, Finland; 10 Institute of Health Sciences, Center For Life-Course Epidemiology, University of Oulu, Oulu, Finland

Corresponding author:

Tuire Salonurmi, PhD

Department of Internal Medicine

University of Oulu

PO BOX 5000, FI-90014 Oulu, Finland

Phone: +358-50-3505282

e-mail: tuire.salonurmi@oulu.fi

Supplementary Table 1. Sequences and annealing temperatures of primers used in the RT-qPCR studies.

Gene	Forward primer 5' – 3'	Reverse primer 5' – 3'	Ann°C
Bmp4	AGCCGAGCCAACACTGTGAG	GAGCTCTGCCGAGGAGATCAC	62
Cebpa	ACGGCGGGAACGCAACAACA	GCTTGCGCAGGCGGTCATTG	62
Cebpb	CGCAACCTGGAGACGCAGCA	GGCTCGGGCAGCTGCTTGAA	61
Fto	GCGGAGGAACGAGAGCGGGA	GCTGCCGGCCTCTCGGAAAA	61
Glut4	GAGCTGGTGTGGTCAATACG	GTTCCAGCAGCAGCAGAG	60
Irx3	CACCCCGCCTTCTACCCCTA	TGGGTGAGGGTCATCTTGGTG	60
Pparg	ACTCCCTCATGGCCATTGAG	TGAGACATCCCCACAGCAAG	61
Rxra	CACCTGCCGAGACAACAAG	GTTCTCATTCCGGTCCTTGC	60
Ucp1	TGCCTCACTCAGGATTGG	GCTTGCATTCTGACCTTCAC	63

Fto

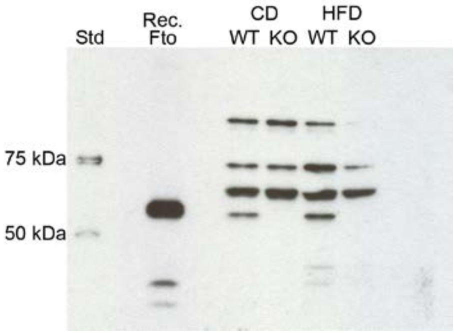

Supplementary Figure 1. Western blot of mouse epididymal WAT proteins after Fto antibody treatment. *WT* wild type; *KO Fto*-knockout; *CD* control diet; *HFD* high-fat diet. According to the manufacturer, the correct band size for Fto is 60 kDa.

Adiponectin

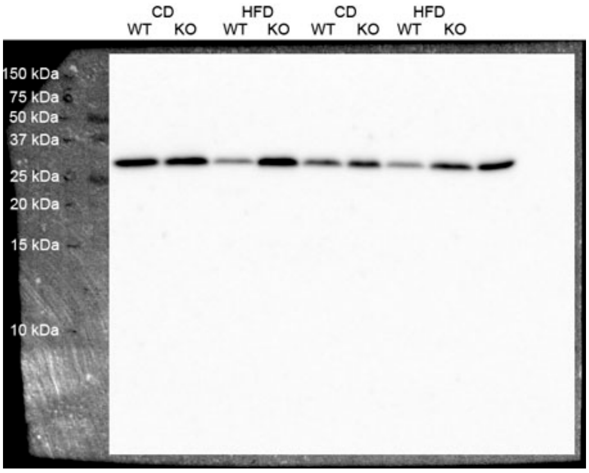

Leptin

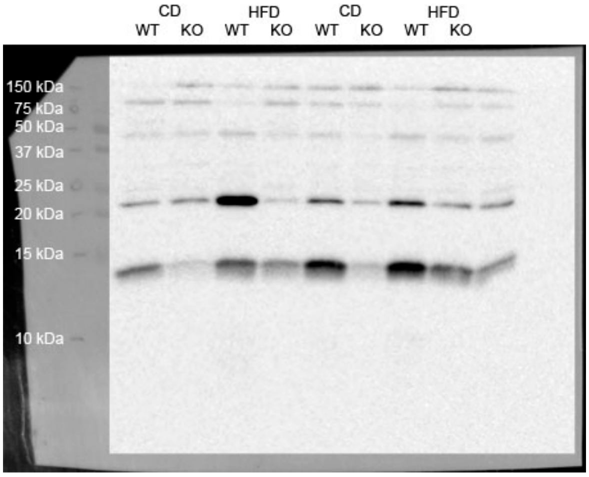

Gapdh

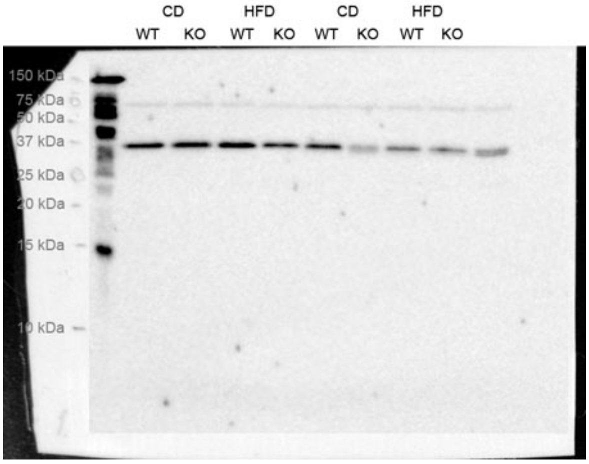

Supplementary Figure 2. Western blot of mouse epididymal WAT proteins after adiponectin, leptin and Gapdh antibody treatment. *WT* wild type; *KO Fto*-knockout; *CD* control diet; *HFD* high-fat diet. According to the manufacturer, the correct band sizes are 30 kDa for adiponecin, 16 kDa for leptin and 37 kDa for Gapdh. Same membrane is shown for each antibody.
